# Supplementary material for: Association between oral microbiome and seven types of cancers in East Asian population: a two-sample Mendelian randomization analysis
Source: Front Mol Biosci. 2023 Nov 21;10:1327893. doi: 10.3389/fmolb.2023.1327893 (PMC10702768; doi:10.3389/fmolb.2023.1327893)
Supplement: Supplementary file 1 [file DataSheet1.ZIP › Supplementary Materials/MR plots for tongue/tongue═╝/Prostate cancer/pheno.1066_to_prostate cancer_scatter.pdf]

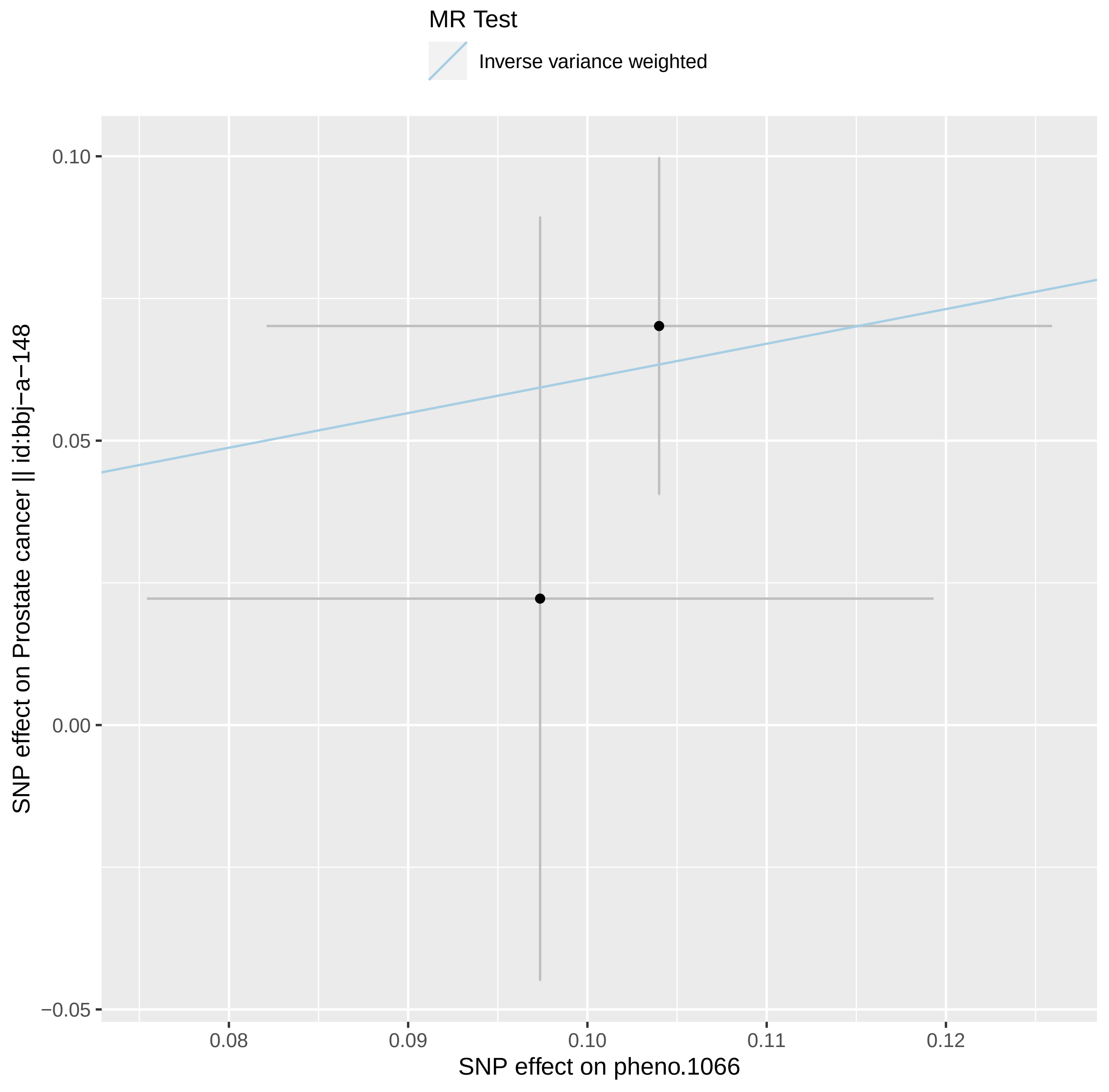
MR Test
Inverse variance weighted
0.10
0.05
0.00
−0.05
0.08
0.09
0.10
0.11
0.12
SNP effect on pheno.1066
SNP effect on Prostate cancer || id:bbj−a−148
